# Breaking a dogma: orthodontic tooth movement alters systemic immunity

**DOI:** 10.1186/s40510-024-00537-z

**Published:** 2024-10-07

**Authors:** Yehuda Klein, Eilon David, Noy Pinto, Yasmin Khoury, Yechezkel Barenholz, Stella Chaushu

**Affiliations:** 1 Department of Orthodontics, Faculty of Dental Medicine, Hebrew University of Jerusalem, Hadassah Medical Center, Jerusalem, Israel; 2 Department of Biochemistry, Institute for Medical Research Israel-Canada, Hebrew University-Hadassah Medical School, Jerusalem, Israel

## Abstract

**Background:**

The prevailing paradigm posits orthodontic tooth movement (OTM) as primarily a localized inflammatory process. In this study, we endeavor to elucidate the potential ramifications of mechanical force on systemic immunity, employing a time-dependent approach.

**Materials and methods:**

A previously described mouse orthodontic model was used. Ni-Ti. springs were set to move the upper 1st-molar in C57BL/6 mice and the amount of OTM was. measured by µCT. Mice were allocated randomly into four experimental groups, each. corresponding to clinical phases of OTM, relative to force application. Terminal blood. samples were collected and a comprehensive blood count test for 7 cell types as well as. proteome profiling of 111 pivotal cytokines and chemokines were conducted. Two controls. groups were included: one comprised non-treated mice and the other mice with inactivated springs.

**Results:**

Serum immuno-profiling unveiled alterations in cellular immunity, manifesting as. changes in percentages of leukocytes, monocytes, macrophages, neutrophils, and. lymphocytes, alongside key signaling factors in comparison to both control groups. The systemic cellular and molecular alterations triggered by OTM mirrored the dynamics previously described in the local immune response.

**Conclusions:**

Although the exact interplay between local and systemic immune responses to orthodontic forces require further elucidation, our findings demonstrate a tangible link between the two. Future investigations should aim to correlate these results with human subjects, and strive to delve deeper into the specific mechanisms by which mechanical force modulates the systemic immune response.

**Supplementary Information:**

The online version contains supplementary material available at 10.1186/s40510-024-00537-z.

## Introduction

Orthodontic tooth movement (OTM) is enabled by a mechanically induced immune reaction which, in turn, triggers bone remodeling [1]. The biological processes underlying bone remodeling involve cellular changes that are modulated by chemical messengers, released from immune cells [2, 3]. Studies revealed that immune cells, such as neutrophils, monocytes, macrophages, NK, mast, basophils, eosinophils, and even adaptive immune cells, such as T, B and γδT Cells, play a role in OTM [4–6]. On the molecular level, OTM has been proved to induce a significant number of differentially expressed genes associated with both innate and adaptive immune arms, in a time dependent manner [7]. Traditionally, OTM is divided into 4 phases: initial, arrest, acceleration and linear [4, 8, 9]. Chaushu et al. recently summarized the cellular and molecular processes underlying each phase [4].

Currently, the accepted dogma is that the OTM associated “sterile” inflammation is restricted to the local area surrounding the moving tooth and has no systemic effects.

However, studies showed that local inflammatory processes, such as those found in periodontal disease trigger elevations in serum inflammation markers [10–13]. Therefore, it is logical to hypothesize that the OTM related immune reaction is not restricted to the oral tissues. A search in the literature found only a few reports on the potential systemic effects of OTM. Keeling et al. reported that serum acid phosphatase (TRAP) and alkaline phosphatase (ALP) peak in the systemic circulation before their detection in the alveolar bone, preceding the histological changes [14]. Tang et al. identified systemically detectible bone resorption markers in orthodontic patients, including elevated serum TRACP 5b isoform at 2 months post force initiation [15], whereas Zeng et al. showed systemic alterations in circulatory and spleen monocytes [16]. Bilgic et al. revealed variations in C-reactive protein (CRP) levels and complete blood count (CBC) parameters [17], in contrast to MacLaine et al., who reported that the systemic levels of CRP, TNF-a and IL-6 were unaffected [18]. Kloukos et al. recently documented a limited and transient decrease in white blood cell (WBC) count on day 5, followed by recovery by day 14. The CRP levels remained unchanged. [18]

The objective of this study was to explore the systemic immune changes triggered by OTM, using a mouse orthodontic model. We aimed to investigate how the mechanical force affects both cellular and molecular systemic immunity throughout the different phases of OTM. The results contribute significant insights into the systemic impact of OTM, an area which is presently inadequately understood.

## Materials and methods

*Animals and ethics*. The study was approved by the IACUC of the Hebrew University (MD-20-16193-3) and conforms to the ARRIVE guidelines. C57BL mice (male 8-9-week-old) were purchased from Harlan (Jerusalem, Israel). Only male mice were used in this study, to avoid the effect of the sex cycle and hormonal changes. The animals were housed in the Specific Pathogen-Free Facility of the Hebrew University, at 25 °C with a 12/24 h light/dark cycle and were fed a granular diet. Body weight and health were monitored every other day.

### OTM mouse model (Fig. 1A)

The OTM mouse model was performed as described elsewhere [7, 19]. Briefly, following anesthesia, a transverse hole was drilled through both maxillary incisors at the apical third of the-crown using a drilling bur, and a stainless-steel ligature wire was inserted through the hole. A non- absorbable Ethilon 5 − 0 wire was inserted beneath the contact point of the left first and the second molars in the maxillae. Then, the 3 mm nickel-titanium closed coil spring (TOMY International, Tokyo, Japan) was connected to the Ethilon wire and tightened to the upper left 1st molar (ULM1). From its second edge, the spring was connected to the stainless-steel wire, thus, the spring was activated and generated a constant force of a mesial tipping movement of ULM110g for 14 days. During the experiment, the mice were monitored daily for appliance detachment and clinical condition. A total of 70 C57BL 8-week old male mice were randomly divided into three experiments of OTM:

**Fig. 1:**
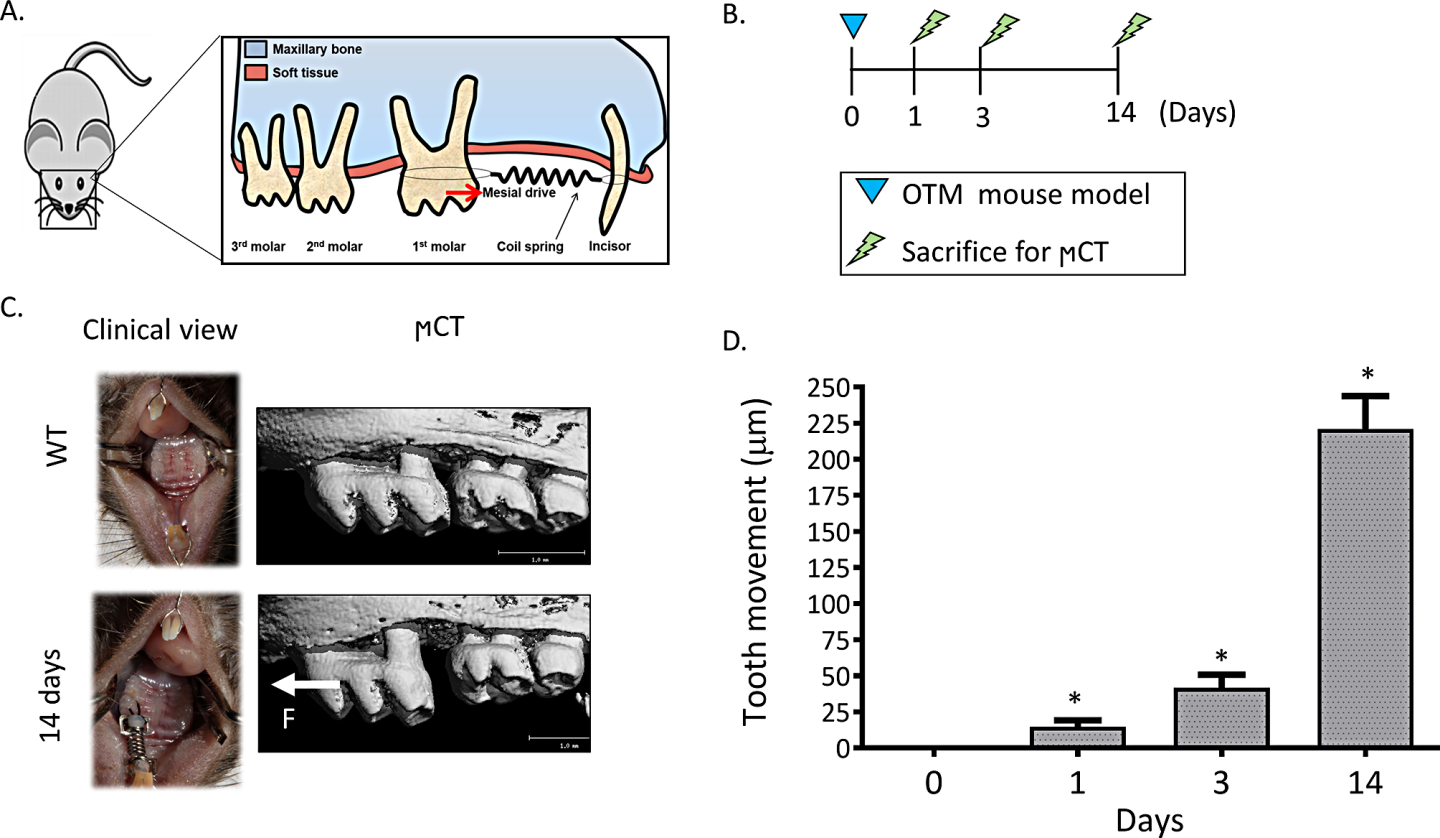
OTM mouse model. (**A**) Illustration of the OTM mouse model, (**B**) Timeline of the experiment. OTM was initiated at day 0 and mice were sacrificed at 3 different timepoints for µCT analysis (*N* = 5/group). (**C**) Representative clinical and 3D images at the end of OTM (day 14), (**D**) The amount of OTM (µm) at the different timepoints

#### Experiment 1

20 mice (*N* = 5/group) were sacrificed at 0, 1, 3, and 14 days post-force application, and the amount of OTM was analyzed by µCT.

#### Experiment 2

25 mice (*N* = 5/group) were sacrificed at 0 (WT), 1, 3, 6, and 14 days post-force application, and blood was collected for a complete blood count (CBC) of the immune cells.

#### Experiment 3

20 mice (*N* = 5/group) were sacrificed at 0 (WT), 1, 3, and 14 days post-force application, and blood was collected for serum proteome profiling.

In addition, a control group with an inactive spring was included and compared to the day 3 active spring group from experiment 2 (*N* = 5). This control group was used to determine whether the observed changes are attributed to OTM rather than the inflammatory reaction caused by the presence of the coil-spring.

#### Sample preparation

For µCT analysis: mice were anesthetized and sacrificed and the maxillae harvested. Maxillae were immersed in 4% paraformaldehyde (PFA) (pH 7.4) in phosphate-buffered-saline (PBS) (at 4 °C) for 4 h for fixation of the tissue, and then kept in 70% Et-OH (ethanol).

For complete blood count: under anesthesia, blood was collected directly from the heart via terminal cardiac puncture using a 25G needle inserted through the rib cage. Blood samples were collected in lithium-heparin tubes and analyzed within 4 h of collection in Bet-Dagan Veterinary Hospital with the aid of blood cell counter machine (Advia 2120i, Siemens, Erlangen, Germany).

For proteome profiler analysis: following terminal blood collection via heart puncture, blood samples were centrifuged (700 rpm, 10 min, 4^0^) for RBC/serum separation and sera were collected.

#### µCT analysis

Samples were scanned with µCT scanner (µCT40^®^, SCANCO, Switzerland) at 10 μm isotropic nominal resolution, 70 kV energy, 114µA intensity, and 1000 projections at 200 ms integration time. Two- and three-dimensional images were constructed. The amount of OTM was measured as the distance between the height of contours of the first and the second left maxillary molars, as previously described (Klein et al. 2018). The timeline of this experiment is illustrated in Fig. 1B.

### Proteome Profiler and bicinchoninic acid (BCA)

Sera samples were mixed with Protease and Phosphatase inhibitor cocktail (Sigma) and the total protein concentrations were first measured using BCA assay (Thermo Scientific©). Following protein normalization, a pool for each group was prepared. For secretome profiling, we used the Proteome Profiler™ Mouse XL cytokine Array (R&D systems^®^), according to manufacturer instructions. Analysis was conducted using the “Image Lab” software, which quantified the protein amount based on the size and intensity (pixel density) of each dot [20]. The proteins were categorized into chemokines, cytokines, growth factors, complement system components, tissue remodeling factors, immune cell markers, angiogenesis factors, coagulation proteins, adhesion molecules, IGFBPs, and CXCLs (as illustrated in Supplementary Fig. 2).

### *Statistical analysis*

All analyses were performed with GraphPad Prism v.8 (GraphPad Software, San Diego, CA, USA), and the numerical values obtained are expressed as means ± SEM. Normally distributed data were analyzed using Student’s one-tailed unpaired t-test (assuming equal variances). A minimum of 5 animals per group was required to achieve a power analysis > 0.8. Statistical significance is indicated by the asterisk symbols: *p < 0.05, **p < 0.01, ***p < 0.005.

## Results

### OTM measurements

The amount of OTM of the first molar was 15 ± 4, 43 ± 7 and 217 ± 18 μm at 1, 3 and 14 days post force initiation, respectively (Fig. 1C, D).

### Immune cellular changes in mice sera, in response to OTM

**Fig. 2:**
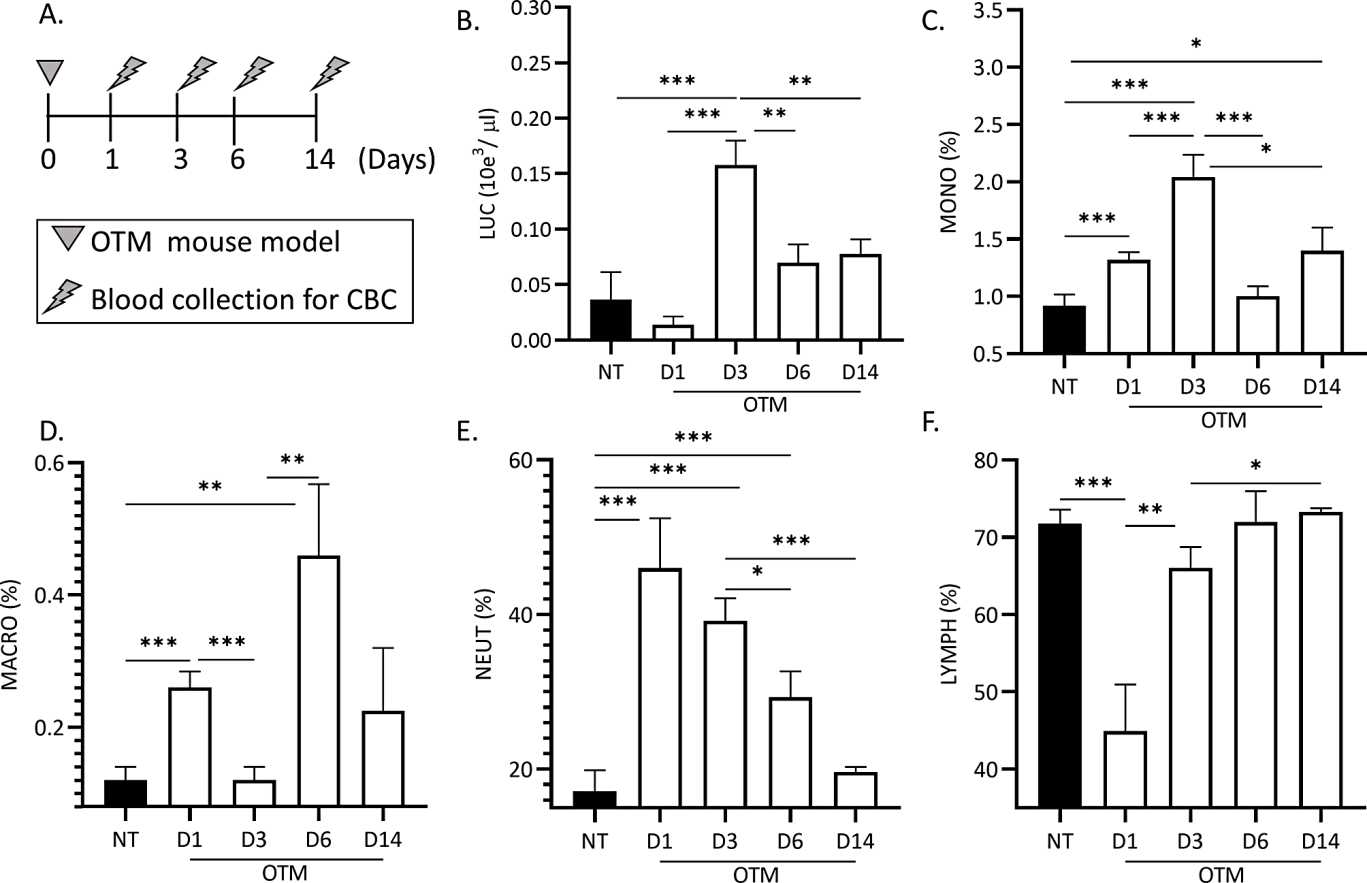
Dynamics of immune cells’ changes in mice sera in response to OTM (CBC = complete blood count. OTM = orthodontic tooth movement). (**A**) Experimental timeline (*N* = 5/ group). (**B**) Leukocyte numbers (10e3/ µl). (**C**) Monocyte percentage (%). (**D**) Macrophage percentage (%). (**E**) Neutrophil percentage (%). (**F**) Lymphocyte percentage (%)

Figure 2 illustrates the dynamics of the immune cellular changes in sera, in response to OTM. OTM treated mice were sacrificed at days 1,3,6 and 14 post force initiation. (Fig. 2A.).

A significant increase in the percentage of leukocytes, monocytes, neutrophils and lymphocytes (*p* = 0.001, *p* = 0.0006, *p* = 0.03, *p* = 0.009 respectively, Fig. 2) and a slight, non-significant increase in macrophages were observed in the OTM treated group compared to the control group with inactivated springs (Figure S1).

Sera immuno-profiling revealed alternations in leukocytes, monocytes, macrophages, neutrophils, and lymphocytes numbers/percentages during the different phases of OTM, in comparison to untreated WT mice.

OTM significantly increased leucocyte numbers at d3 compared to WT mice and d1 (0.158 ± 0.043 10e^3^/µl versus 0.036 ± 0.055 10e^3^/µl and 0.014 ± 0.014 10e^3^/µl, respectively, *P* < 0.003, Fig. 2B). A gradual dropdown back to baseline values was observed at d6 and d14 (0.07 ± 0.03 10e^3^/µl and d14 = 0.078 ± 0.025 10e^3^/µl, respectively, *P* = 0.006).

The monocyte percentage out of total leukocytes significantly increased at d1 and d3 in OTM treated in comparison to WT mice (1.32 ± 0.132, *p* = 0.004 and 2.04 ± 0.39%, *p* = 0.0004 versus 0.92 ± 0.19%, respectively) followed by a significant dropdown at d6 and day14 (Fig. 2C).

The macrophage percentage out of total leukocytes significantly increased at d1 compared with WT mice (0.12 ± 0.04% vs. 0.26 ± 0.04%, respectively, *p* = 0.001) but dropped (0.012 ± 0.04%) at d3 compared to d1 (*P* = 0.001). Interestingly, at d6 macrophage percentage peaked to 0.46 ± 0.21%, a statistically significantly higher percentage than d3 (*p* = 0.007). At d14 macrophage percentage returned to baseline (0.22 ± 0.16%) (Fig. 2D).

The neutrophil percentage out of total leukocytes significantly peaked at d1 compared with WT mice, reaching peak levels of 46 ± 12.8% versus 17.16 ± 5.41%, respectively (*p* = 0.04). Subsequently, the neutrophil percentage gradually decreased between d3, d6 and d14 and returned to baseline levels (39.2 ± 5.7%, 29.32 ± 6.6%, 19.6 ± 1.28%, respectively) (Fig. 2E).

The lymphocyte percentage out of total leukocytes significantly decreased from baseline levels of 71.7 ± 3.6% to 44.9 ± 12.08% at d1, *p* = 0.0013. During the following days a gradual increase was recorded: 66.02 ± 5.43% at d3, 71.94 ± 8.08% at d6 up to normal values of 73.3 ± 0.9% at d14. (Fig. 2F.)

### Sera proteome alternations in response to OTM

**Fig. 3:**
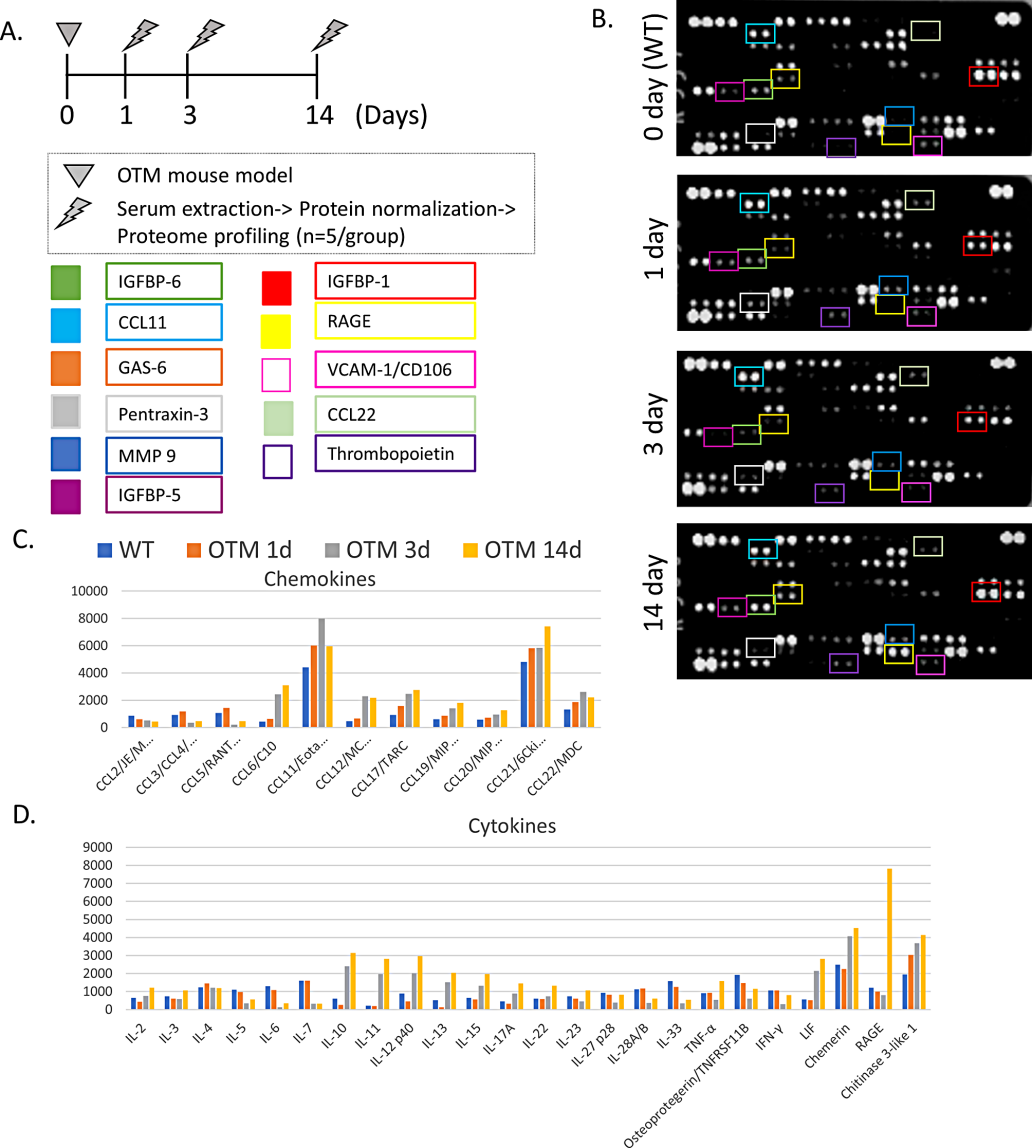
OTM influences the composition of the circulatory secretome throughout the various phases of OTM, (**A**) Experimental timeline: OTM model was performed, and mice were sacrificed at days 1, 3, and 14 with terminal blood collection. The collected sera were utilized for protein normalization, followed by proteome profiler analysis (*N* = 5 per group). (**B**) Array images of the 4 membranes showing positive signals seen on developed films. Quantification of protein levels was performed by pixel density measurements. The pooled samples were combined based on their respective groups (WT, OTM-d1, OTM-d3, OTM-d14). Proteins were subsequently subcategorized for analysis: **C**. Chemokines. **D** Cytokines

To validate the CBC results and explore the association between the cellular and molecular immune changes, we next performed a proteome profiler assay, which enables the detection of 111 extracellular pivotal signaling molecules in each experimental group (WT, d1, d3, and d14, Fig. 3A, B).

Our analysis revealed significant differences, especially in the expression of cytokines and chemokines. OTM led to an increase in the expression of certain pro-inflammatory cytokines at d14 of post OTM, including IL-2, IL-12, IL-17 A, IL-15, TNF-a, and RAGE, as well as an increase in the expression of anti-inflammatory cytokines such as IL-10, IL-11, and IL-13 at d3 or d14. Some dual-function cytokines, such as Leukemia Inhibitory Factor (LIF), chemerin, and Chitinase 3-like-1, were also increased after 3–14 days of OTM (Fig. 3C & D).

Additionally, chemokines were notably affected, with OTM inducing systemic changes in crucial chemokines like CCL6, CCL11, CCL19, CCL21, CXCL 9, 10, and 11. Furthermore, alterations were observed in other growth factors, tissue remodeling factors, immune cell markers, angiogenesis factors, adhesion molecules, and IGFBP’s (Figure S2).

**Fig. 4:**
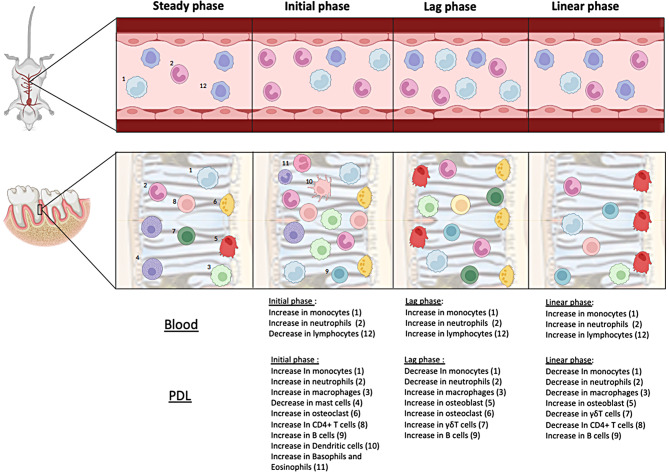
Schematic illustration of the main immune cells involved in each orthodontic tooth movement phase, in blood and PDL (based on Chaushu et al. JDR). Yechezkel Barenholz– conceptualization, methodology, writing - Review & Editing and Resources. Stella Chaushu- Contributed to the conceptualization, methodology, investigation, validation, writing - Review & Editing, supervision, project administration and funding acquisition. SC takes responsibility for the integrity of the data analysis. All authors gave their final approval and agreed to be accountable for all aspects of the work

The schematic illustration portrays the concurrent changes in immune cells in the periodontal ligament (PDL) (based on Chaushu et al. JDR) [4] and systemic circulation during the different phases of OTM. (Fig. 4).

## Discussion

The present study represents the first comprehensive analysis of systemic immune cellular and molecular changes, elicited by orthodontic force. Utilizing an orthodontic mouse model offered a more controlled experimental environment compared to human studies, which are susceptible to variability stemming from genetic disparities, environmental factors (e.g., oral hygiene status), and other variables [21].

The experimental group was divided into 4 subgroups corresponding with the clinical phases of OTM, recently described in detail by Chaushu et al. [4]: the initial phase − 24 h (d1), the lag phase − 3–6 days (d3, d6) and the acceleration and linear phase − 14 days (d14), post force initiation. Sera were collected for CBC and proteome profiling to detect changes in immune cell types, as well as cytokines, chemokines, growth factors, adhesion molecules and more.

In contrast to periodontitis, OTM is considered a sterile and non-pathogenic process, where local immune activation is mediated mechanically, rather than by bacteria [22]. However, studies have suggested that orthodontic fixed appliances can provide a rough surface that enhances the adhesion of oral bacteria, leading to dental plaque accumulation and subsequent inflammatory responses [23, 24]. Additionally, the orthodontic tooth movement mouse model involves upper jaw injury to fixate the NiTi closed coil spring, which might trigger a systemic response and introduce potential bias. To isolate the mechanically induced systemic inflammatory response, we examined changes in circulatory immune cells in activated versus inactivated springs at d3 of OTM, as previous data indicated that the most significant molecular changes occur at this time point [7]. The results illustrate a fourfold increase in the total number of circulatory leukocytes in mice with activated versus inactivated springs. Notably, the inactivated spring group demonstrated differences in immune cell percentages compared to WT mice without an appliance and surgery, suggesting that the surgery and wearing of the appliance, which accumulates plaque, induce minor immune changes as well. Nevertheless, we showed here that the application and activation of the force is the important causative factor for systemic alterations in immune cell expression. Subsequently, we characterized the systemic alterations in a time dependent manner.

## The initial phase

The initial OTM phase commences immediately upon force application and spans up to 3 days. The immediate tooth movement primarily results from intraosseous tooth displacement within the alveolar socket [4, 9]. During this phase, orthodontic forces stimulate vasodilatation and extravasation of leukocytes from capillaries, leading to an acute inflammatory reaction primarily composed of neutrophils and monocytes, peaking at day 3 [4, 5, 7, 25]. Activated neutrophils play a role in removing tissue debris and secreting chemotactic mediators, which attract monocytes and macrophages. Neutrophils also promote osteoclast differentiation and bone resorption through upregulation of RANKL [26].

In the present study we observed a significant increase in circulating neutrophils, with the peak occurring 24 h after the onset of OTM, suggesting a potential temporal association between circulatory and PDL neutrophils, possibly due to the extravasation of neutrophils from the bloodstream into the PDL and adjacent alveolar bone. Concurrently, we observed increased systemic expression of CCL11, CCL19, CCL21, E-Selectin, P-Selectin, and C5a, all of which are involved in the chemotaxis, adhesion and activation of neutrophils and leukocytes [27–32].

During OTM, neutrophils secrete chemotactic mediators that attract monocytes and macrophages to the PDL [5]. Monocytes serve as important precursors for macrophages and osteoclasts, with macrophages being capable of further differentiation into osteoclasts [33, 34]. Studies have also shown that macrophages play a crucial role in initiating OTM, as they can differentiate into the proinflammatory M1 type [35]. This polarization toward the M1 macrophage subtype is critical for bone resorption and the initiation of OTM [36]. Klein et al. demonstrated significant upregulation of phagocytosis pathways in macrophages and monocytes, further confirming their participation in OTM [7]. Our results indicate a significant increase in monocyte and macrophage percentages in circulation during the initial phase of OTM, paralleling other studies which demonstrated an increase in circulating monocytes during infection or sterile inflammation [37, 38]. This contrasts with Zeng et al., who showed that the percentages of monocytes exhibited an early decline followed by subsequent recovery in both the blood and spleen [16].

Notably, circulatory macrophage percentages significantly increased on d1, followed by a marked decrease at d3, retuning to baseline levels. This systemic decrease could be attributed to the depletion of circulatory macrophages due to their extravasation into the PDL, where they further differentiate into osteoclasts or M1 macrophages [33, 35]. Compared to the inactive spring group, the percentage of macrophages on day 3 in the OTM group was not significantly different. Nevertheless, it is evident from the immune profile of other immune cells that the activation of force itself, rather than injury or plaque accumulation, causes systemic changes in the immune system.

The increased systemic expression of IL-15, IL-17 A, CXCL 9, 10, 11, and CCL6/C10, which are essential chemokines and cytokines associated with the migration and activation of monocytes and macrophages, could account for the differential systemic expression of these cells [39–43].

The expression of B, CD4 + T, and NK lymphocytes in the PDL is increased during the initial phase [4, 7]. Their involvement in OTM has been proved in many previous studies. T-cell-deficient immunocompromised mice showed reduced OTM [6], conditional ablation of γδT cells diminished OTM [5] and depletion of NK cells inhibited OTM [4]. Furthermore, increased numbers of CD4 + T cells, enhanced expression of T-cell costimulatory molecules CD40 and CD40 ligand, and an increase in Th1 and Th17 cells in response to mechanical force, further emphasize their role [33, 44, 45]. Kook et al. observed an immediate increase in CD220 + B cells in the PDL, highlighting B cell involvement [33]. B and T lymphocytes produce proinflammatory cytokines that stimulate RANKL secretion, promoting osteoclastogenesis and bone resorption [2]. The initial drop in circulatory lymphocytes on d1 observed in this study may be attributed to system-to-region redistribution, followed by recovery in the following days, eventually returning to steady-state values. Elevated plasma levels of chemotactic factors, including C5a, CCL6, CCL11, CCL19, CCL21, CXCL 9,10, and 11, may contribute to the recruitment of T and B lymphocytes to the inflammatory site. The serum expression of CD40 is also increased during this phase, highlighting its significance in CD4 + T-cell activation and function during OTM [44, 46].

### The lag phase

The lag phase, which lasts between 3 and 7 days in mouse models [47] is associated with hyalinization of the PDL in the pressure area, resulting in no tooth movement until the hyalinized tissue is removed [9, 48]. This phase involves the occlusion of blood vessels leading to cell death, followed by the resorption of hyalinized tissue by macrophages and the undermined resorption of the lamina dura by osteoclasts. Complete resorption of the lamina dura ultimately facilitates the resumption of tooth movement [49]. During this phase, a resolution of acute inflammation occurs to prevent irreversible tissue damage. The resolution of inflammation is characterized by the cessation of neutrophil influx, the removal of necrotic tissue debris by macrophages, and a reduction in monocyte expression [4, 5, 50]. Specifically, in the PDL, the lag phase is characterized by a general decrease in leukocytes, particularly neutrophils, monocytes, Th1, and Th17, and an increase in M1 macrophages, B cells, Treg, and γδ-T cells [4, 5, 7, 44]. Accordingly, we observed that circulatory leukocytes peaked at the beginning of the lag phase (d3) but then gradually decreased (d6) to steady state levels as the resolution of inflammation advanced. Among the leukocytes, neutrophils demonstrated a similar trend. They reached their peak on d1, but significantly decreased by d3 and d6, aligning with the cellular reaction in the PDL during the lag phase. Simultaneously, the systemic percentages of lymphocytes and macrophages, possibly the M1 subtype, gradually increased during this phase, reaching peak levels at d6.

The percentage of monocytes peaked on d3 but then drastically decreased by d6, returning to steady state levels. The systemic expression mirrored the local expression of monocytes in the PDL and alveolar bone during OTM, namely a peak expression on d3 with a subsequent decrease during the lag phase [4, 5].

A decrease in specific pro-inflammatory cytokines (IL-5, IL-6, IL-7, TNF-a, and IFN- g), along with an increase in anti-inflammatory cytokines (IL-10, IL-11, IL-13), may explain the decreased number of tissue and circulatory neutrophils and the partial resolution of inflammation in the lag phase [51–53]. IL-10 plays a vital role in the anti-inflammatory process during OTM by promoting tissue debris removal, inhibiting bone resorption and tissue repair, and inducing osteoblastogenesis, ultimately leading to an overall anabolic effect [53]. Furthermore, an elevated systemic expression of IL-15, IL-17, Chemerin, CCL6, CCL11, CCL19, CCL21, CXCL 9, 10, and 11, M-CSF, GM-CSF, E-selectin, P-selectin, and C5a was also detected. These factors are linked to chemotactic activity or the induction of differentiation in monocytes, macrophages, and lymphocytes [25, 27–31, 41–43, 54–57].

The lag phase is further marked by enhanced osteoclasts and osteoblasts activity, evident through increased RANKL expression and osteoid deposition [4, 58]. The increased expression of IL-11, IL-17, CCL11, CCL19, CCL21, HGF, M-CSF, ICAM-1, CXCXL9, CXCL10 and Amphiregulin and the decreased expression of IL-33, IFN-g and OPG is associated with enhanced bone metabolism characteristic of this phase [5, 27, 28, 59–70].

### The acceleration and linear phases

The resolution of undermining resorption in the lag phase leads to accelerated tooth movement, followed by a linear movement phase that persists as long as constant force is maintained [9, 71]. During these phases, there is a shift towards homeostasis, characterized by upregulated pathways associated with cell proliferation, migration, cytoskeleton rearrangement, wound healing, and angiogenesis. These pathways indicate that tissues are actively attempting to return to a state of equilibrium. Additionally, the upregulation of pathways linked to macrophages and osteoblasts is evident [7].

At the OTM site, there is a reduction in the expression of M1-like macrophages, neutrophils, monocytes, γδ cells, and CD4 + T cells, while CD3 + and B cells increase [4, 5]. Our current results show a similar pattern in the blood with a decrease in the percentage of neutrophils and macrophages compared with the lag phase, while circulatory monocytes show insignificant increase compared to the lag phase, ultimately returning to baseline levels as determined in WT mice. Additionally, there is an increase in the percentage of lymphocytes on d14, possibly attributed to the increased expression of B cells.

The rise in various chemokines and cytokines, such as IL-11, IL-12, IL-13, IL-15, CCL19, and CCL21, may lead to the increased numbers of lymphocytes in the bloodstream, promoting their differentiation, proliferation, activation, and migration [29, 72–75]. This phase, marked by extensive bone remodeling, is also characterized by increased systemic expression of factors associated with osteoblastic and osteoclastic activity, including IL-2, IL-3, IL-10, IL-11, IL-17, LIF, CCL11, CCL19, CCL21, M-CSF, CXCL9, CXCL10, and Amphiregulin [5, 27, 59, 60, 63, 66–70, 76, 77]. Notably, the inflammatory response indicator RAGE was significantly upregulated during the linear phase, confirming the presence of a systemic inflammatory reaction in response to OTM [77, 78].

Some limitations arising from the methodology of this study are acknowledged. One limitation is that animal studies might not fully reflect the situation in humans. Although animal studies are more predictable and reproducible, the results should be interpreted with caution. Another limitation relates to the ratio between the mechanical force and the body weight, which was much higher in animal models in comparison to humans.

## Conclusion

Our study uncovered that orthodontic forces induced systemic changes far beyond the OTM site. These cellular and molecular systemic alterations mirror the dynamic responses observed locally. These findings call for further research to confirm if these results are reproducible in human subjects and to unravel the intriguing mechanisms by which mechanical force shapes the systemic immune response.

## Electronic supplementary material

Below is the link to the electronic supplementary material.

Supplementary Material 1

Supplementary Material 2

Supplementary Material 3

## Data Availability

No datasets were generated or analysed during the current study.

## Abbreviations

OTM: Orthodontic tooth movement
PDL: Periodontal ligament
WBC: White blood cell
CRP: C-reactive protein
CBC: Complete blood count
