# Supplementary figures and images for: Breaking a dogma: orthodontic tooth movement alters systemic immunity
Source: Prog Orthod. 2024 Oct 7;25:38. doi: 10.1186/s40510-024-00537-z (PMC11456555; doi:10.1186/s40510-024-00537-z)

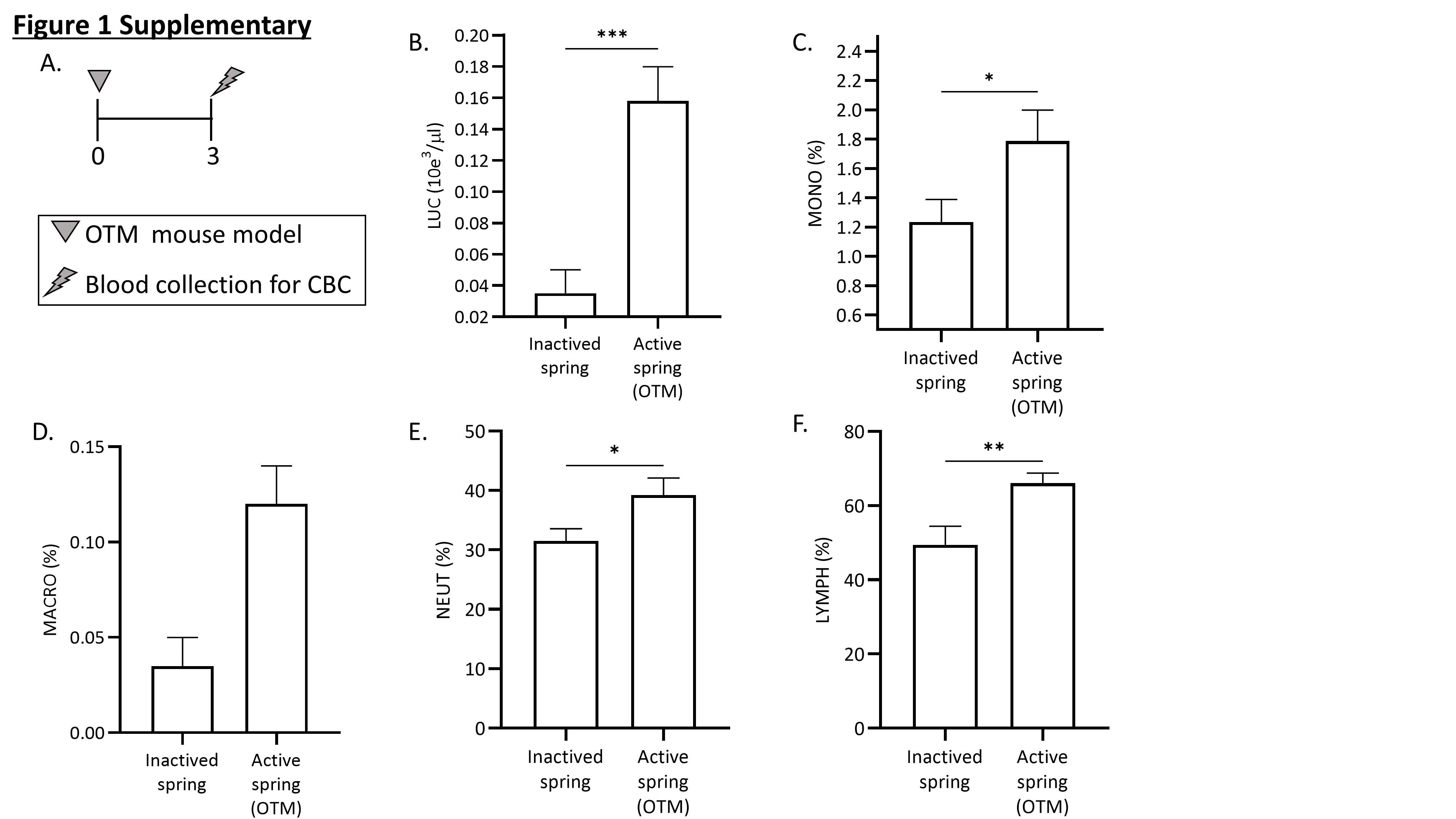

Supplement: Supplementary file 1 — Supplementary Material 1 [file 40510_2024_537_MOESM1_ESM.tif]

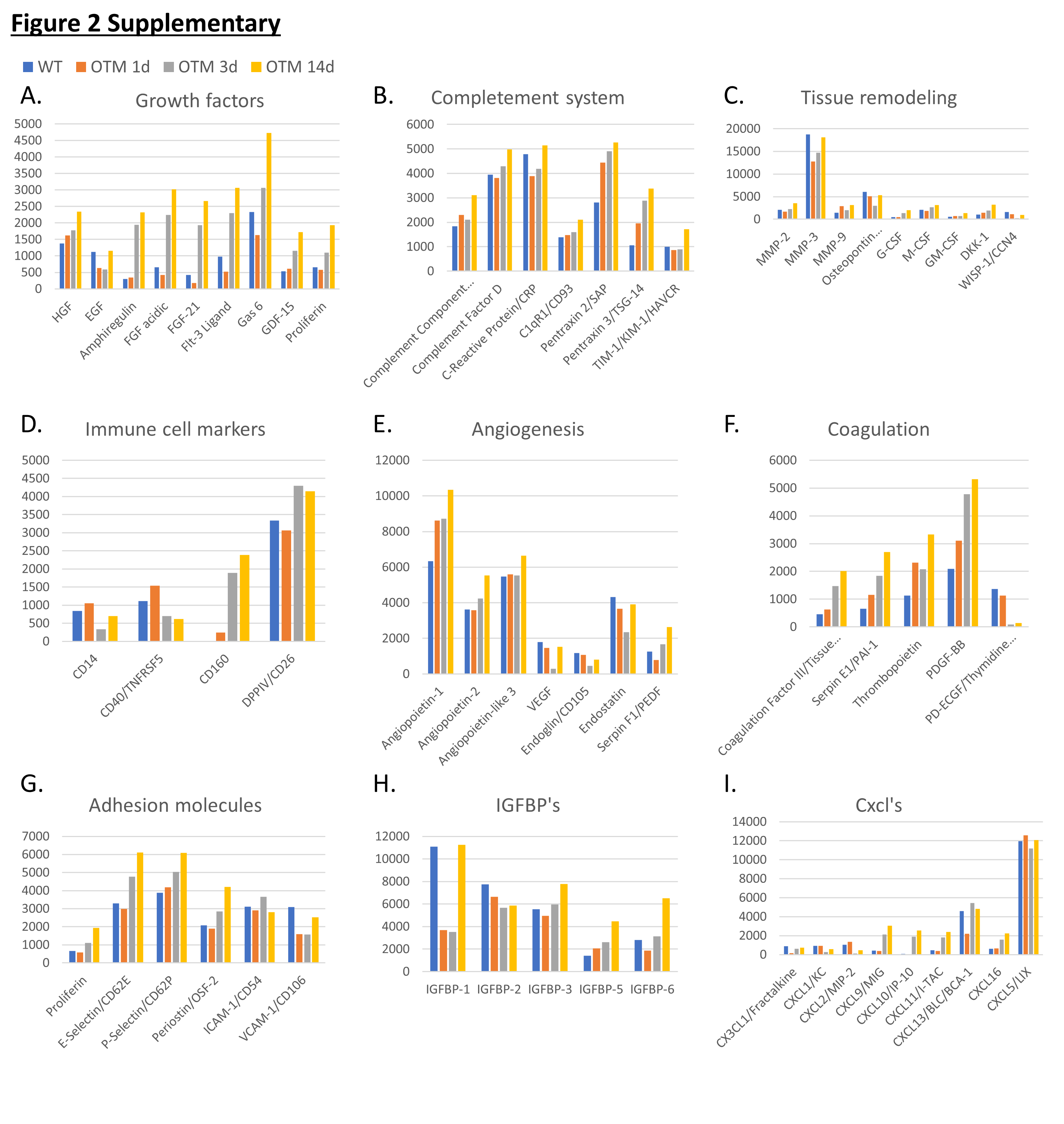

Supplement: Supplementary file 2 — Supplementary Material 2 [file 40510_2024_537_MOESM2_ESM.tif]
