# Supplementary material for: Breaking a dogma: orthodontic tooth movement alters systemic immunity
Source: Prog Orthod. 2024 Oct 7;25:38. doi: 10.1186/s40510-024-00537-z (PMC11456555; doi:10.1186/s40510-024-00537-z)
Supplement: Supplementary file 3 — Supplementary Material 3 [file 40510_2024_537_MOESM3_ESM.docx]

**Figure 1 supplementary.**

**The immune cellular changes in mice sera, in response to inactivated versus activated springs at day 3.**

A. Experimental timeline: All mice were sacrificed at d3 with terminal blood collection for CBC (n=5/ group)

B. Leukocytes (10e3/ μl)

C. Monocytes (%)

D. Macrophages (%)

E. Neutrophils (%)

F. Lymphocytes (%)

**Figure 2 supplementary.**

**OTM affects the circulatory secretome composition during the different phases of OTM.**

A. Growth factors

B. Complement system

C. Tissue remodeling

D. Immune cell marker

E. angiogenesis

F. Coagulation

G. Adhesion markers

H. IGFBP's

I. CXCL's
